# Converging Evidence of a Specific Vulnerability of Young Boys to Parental Childhood Trauma

**DOI:** 10.1016/j.jaacop.2025.03.001

**Published:** 2025-04-02

**Authors:** Karl Larouche, Julia Garon-Bissonnette, Roxanne Lemieux, Kim Deschênes, Gabrielle Duguay, Jean-Pascal Lemelin, Nicolas Berthelot

**Affiliations:** a Université du Québec à Trois-Rivières, Trois-Rivières, Quebec, Canada; b Pole of expertise and research on men's health and well-being, Quebec City, Quebec, Canada; c Centre d’études interdisciplinaires sur le développement de l’enfant et la famille, Trois-Rivières, Quebec, Canada; d CERVO Brain Research Center, Quebec City, Quebec, Canada; e Interdisciplinary Research Center on Intimate Partner Relationship Problems and Sexual Abuse, Montreal, Quebec, Canada; f Groupe de recherche et d’intervention auprès des enfants vulnérables et négligés (GRIN), Trois-Rivières, Quebec, Canada; g Vanderbilt University, Nashville, Tennessee; h Université de Sherbrooke, Sherbrooke, Quebec, Canada

**Keywords:** mothers, fathers, childhood trauma, child development, child sex

## Abstract

**Objective:**

Childhood trauma is common among parents and can have intergenerational effects. Preliminary evidence suggests that young boys may be more vulnerable to maternal childhood trauma than girls. This finding needs to be replicated, and it remains to be determined whether it also applies to paternal childhood trauma. The current study aims to examine the associations between parental childhood trauma and 3 indicators of early functioning (general development, socioemotional development, and temperament) in offspring and to assess the moderating role of child sex.

**Method:**

Childhood trauma was assessed during pregnancy in 3 community samples of women (N = 143; N = 195; N = 216) and postnatally in 1 community sample of fathers (N = 165). Child functioning was assessed using parental reports of child development and temperament between 6 and 38 months postnatal. Regression-based moderation analyses were conducted.

**Results:**

Significant associations between parental childhood trauma and adverse child outcomes were observed in all samples. A moderating role of child sex was supported in 3 of the 4 samples, all indicating greater vulnerability to parental childhood trauma among boys.

**Conclusion:**

These findings suggest that maternal and paternal childhood trauma are associated with poorer functioning in infants and toddlers, but only in boys. This has implications for personalized preventive interventions with parents exposed to childhood trauma. These results call for replication with large and diverse samples including biological measures and observational measures of child development.

## Plain language summary

This study utilized three samples of mothers and one of fathers from Quebec, Canada to examine the associations between parental childhood trauma and early childhood functioning. In three of the four samples, young boys exhibited increased vulnerability to parental childhood trauma in comparison to girls. These findings must be replicated to consider their potential for personalized preventive interventions for male offspring of parents exposed to childhood trauma.

Approximately 35% of pregnant women and 28% of fathers-to-be from low-risk samples recruited in the community report having been exposed to at least 1 type of childhood maltreatment in the form of abuse (physical, sexual, or emotional) or neglect (physical or emotional) before the age of 18 years.^1^ When a broader definition of interpersonal trauma that includes other potentially traumatic events shown to have long-term negative associations with mental health (eg, prolonged bullying, witnessing domestic violence, suicide attempts by a parent, persisting role reversal in the parent–child relationship)^2^, ^3^, ^4^ is used, the prevalence of childhood trauma would increase to 1 in 2 expecting parents from low-risk samples.^5^ Previous research, primarily conducted with mothers, shows that parental exposure to childhood maltreatment or adverse childhood experiences (ACEs; which includes maltreatment but also household dysfunction)^6^ has implications for the health and development of their offspring.^7^, ^8^, ^9^, ^10^, ^11^ Recent findings have intriguingly reported that boys may be more vulnerable than girls to the adverse effects of maternal childhood maltreatment^12^ and ACEs^13^ in their early years, but evidence remains scarce. Given the potential implications of this greater vulnerability of boys to parental childhood trauma with regard to intervention and research, this finding needs to be replicated. Considering that both maltreatment^14^ and exposure to other forms of ACEs^15^ would have intergenerational repercussions, we hereafter use the term “childhood trauma” to refer to the broad scope of adverse experiences likely to greatly compromise one’s sense of security and integrity during development.

### Intergenerational Repercussions of Maternal and Paternal Childhood Trauma

The Developmental Origins of Health and Disease model posits that exposure to adverse biological and environmental risk factors during fetal development and infancy may influence early child development, and may exert a profound and enduring impact on an individual’s susceptibility to the development of diverse health and disease outcomes in later life.^16^ Recent studies have paid particular attention to maternal exposure to childhood trauma as a risk factor for poorer maternal health and functioning during pregnancy,^17^^,^^18^ fetal development,^19^ and parenting during the early years of the child,^19^^,^^20^ which, in turn, may negatively influence child development and functioning.^19^^,^^21^^,^^22^ Yet, previous evidence linking maternal childhood trauma to offspring early developmental outcomes are mixed. On the one hand, recent scoping and systematic reviews have reported associations between the accumulation of ACEs in mothers and a higher risk of developmental delays, poor socioemotional development, and a temperament characterized by negative affectivity in their children by preschool age, as well as internalizing and externalizing problems during early childhood, middle childhood, and adolescence.^8^^,^^10^ On the other hand, a recent meta-analysis found small associations between maternal ACEs and internalizing and externalizing difficulties in children 1 to 14 years of age, but no significant associations with early (prior to age 5 years) general and socioemotional development.^15^ There are 2 potential explanations for these inconsistencies. First, many prior studies have overlooked the potential moderating role of child sex.^15^ Second, evidence suggests that childhood trauma extends beyond experiences of abuse or neglect^5^ or the 10 categories assessed by the Adverse Childhood Experiences Study scale.^23^ Notably, additional forms of childhood interpersonal trauma have been shown to significantly enhance our understanding of mental health symptoms in adolescents^23^ and pregnant women.^5^ Therefore, incorporating a broader spectrum of childhood interpersonal trauma (including childhood maltreatment, but also peer victimization, household dysfunction, and deprivation) may enhance the ability to detect significant intergenerational effects of parental childhood trauma.

Far fewer studies have focused on the intergenerational effects of paternal childhood trauma,^8^^,^^11^ despite the important role that fathers play in their child’s life,^24^ the possible epigenetic inheritance of paternal trauma,^25^ and the possibility that childhood trauma alters paternal germ cell line and indirectly influences child development, a central tenet of the Paternal Origins of Health and Disease theory.^26^^,^^27^ The limited evidence suggests that paternal childhood trauma is associated with an increased risk of developmental delays in toddlers^28^ and emotional and behavioral problems in school-aged children and adolescents.^29^^,^^30^ Whereas the moderating effect of child sex in the association between paternal childhood trauma and early offspring development remains greatly overlooked, some evidence from human^31^^,^^32^ and animal^33^ models suggests a sex-specific transmission of early life or pre-conception environmental exposures from fathers to their sons. In addition, to our knowledge, the association between paternal childhood trauma and offspring temperament has never been examined, whereas studies with mothers have shown positive associations between maternal childhood trauma and negative affectivity in offspring,^34^ which is a marker of later behavioral and developmental difficulties.^35^ Studies of temperament, as a specific dimension of infant socioemotional development, appear particularly relevant for several reasons. First, temperament serves as an enduring, biologically rooted foundation of personality^36^^,^^37^ and plays a significant role in developmental psychopathology.^38^ Second, temperament is distinct from other aspects of socioemotional development in terms of its biological underpinnings.^39^^,^^40^ Indeed, research indicates that from 20% to 60% of the variability in temperamental dimensions can be attributed to genetic factors.^41^ Yet, the biological origins of temperament do not preclude environmental influences.^42^, ^43^, ^44^

### Role of Child Sex in the Intergenerational Transmission of Parental Childhood Trauma

Emerging literature suggests that young boys may be vulnerable to parental childhood trauma in a manner different from that of girls. Indeed, researchers observed variations in male and female fetuses’ responses to adverse intrauterine environments, with male fetuses possibly being particularly susceptible to the effects of maternal stress and inflammation during pregnancy.^45^^,^^46^ Postnatal studies also suggest a differential susceptibility to parenting behaviors in boys and girls.^47^^,^^48^ Accordingly, a recent longitudinal study found that maternal childhood trauma, assessed during pregnancy, was directly and significantly related to a clustering of developmental delays in boys aged 3 years or younger, but not in girls.^12^ Similarly, Letourneau *et al.*^13^ found that maternal childhood trauma was linked to internalizing and externalizing behaviors in children aged 2 years or younger via maternal prenatal anxiety and depression, and that this association was moderated by child sex, with boys demonstrating greater vulnerability than girls. However, studies with older children (9-12 years of age) revealed no moderating effect of child sex in the intergenerational repercussions of maternal childhood trauma.^49^^,^^50^ To our knowledge, no studies on the intergenerational effects of paternal childhood trauma have evaluated the moderating effect of child sex.

### Current Study

In the present article, we drew on 4 studies and separate samples (3 samples of mothers and 1 sample of fathers), to pursue 2 primary objectives. First, we assessed the associations between maternal and paternal childhood trauma and 3 domains of functioning in infants and toddlers (general development, socioemotional development, and negative affectivity as a core component of temperament), while controlling for potential confounding variables. The aim of the second objective was to examine whether child sex moderated the associations between both maternal and paternal childhood trauma and 3 indicators of early childhood functioning. We hypothesized that higher levels of parental childhood trauma would be associated with poorer indicators of general development as well as higher levels of socioemotional difficulties and negative affectivity in infants and toddlers. In addition, we hypothesized that boys would be specifically vulnerable to the effect of parental childhood trauma. Given existing evidence that a broad range of childhood interpersonal trauma, and not just childhood maltreatment, is associated with maternal functioning during pregnancy,^5^ which may have implications for offspring development, we used 2 complementary measures of childhood trauma, one assessing childhood maltreatment and the other including a broader range of potentially traumatic experiences. We hypothesized that both measures would be associated with offspring functioning but that stronger associations would be observed using the broader trauma measure.

## Method

### Participants and Procedures

Because a major aim of the current study was to replicate findings on young boys’ greater vulnerability to parental childhood trauma,^12^^,^^13^ we conducted identical analyses on 4 distinct and unrelated samples from Quebec, Canada. Sample 1 includes 143 pregnant women that were recruited during prenatal classes held between July 2015 and September 2018. They completed a series of questionnaires online during their third trimester and again at a follow-up between 10 to 38 months postpartum. Sample 2 includes 195 pregnant women who were recruited during their first prenatal visit, typically around the 12th week of pregnancy, from April 2018 to March 2021. They completed questionnaires online via a secure portal during their second trimester (T1) and between 5 and 14 months postpartum (T2). Sample 3 includes 216 pregnant women who were recruited online through social media (Facebook) during the first COVID-19 mandatory lockdown that occurred in the province of Quebec, Canada, from April 2 to April 13, 2020. Participants completed online questionnaires during pregnancy (T1) and at 6 months postpartum (T2). Sample 4 includes 165 fathers of children aged 3 years or younger who were recruited through a survey panel between July and September 2023. Fathers completed self-administered questionnaires online through a secure portal.

For the 3 studies with mothers, inclusion criteria were being pregnant at the first assessment, being 18 years of age or older, being French speaking, having sufficient reading ability to complete self-report instruments, not having a psychotic disorder, and not reporting preterm birth (<37 weeks), severe peripartum complications, or a congenital disorder in the child at the postnatal assessment. For the study with fathers, inclusion criteria were being 18 years of age and being the biological father of at least 1 child aged 3 years or younger who does not have a neurodevelopmental disorder (eg, autism spectrum disorder) or other chronic, severe, and persistent disorder. All participants provided their consent before participating in the study. The sociodemographic characteristics and descriptive statistics of the main variables for each sample are presented in Table 1.^51^ The samples predominantly include families of White ethnicity with high socioeconomic status, with roughly equal numbers of boys and girls. Institutional review board approval was granted by our university and regional health center.

**Table 1:** Sociodemographic Characteristics of Participants and Descriptive Statistics of the Main Variables in Samples 1 to 4

Sociodemographic characteristics/main variables	Sample 1 (n = 143)			Sample 2 (n = 195)			Sample 3 (n = 216)			Sample 4 (n = 165)		
	% / Mean	n / SD	Range	% / Mean	n / SD	Range	% / Mean	n / SD	Range	% / Mean	n / SD	Range
Age, y	28.58	4.41	17-45	29.63	4.48	18-45	29.67	3.71	20-40	34.08	5.70	21-51
More than 1 child in the household	13.0	14		43.3	84		38.6	83		42.4	69	
Race												
African American	0	0		0.7	1		0.5	1		5.1	8	
Asian	0.7	1		0.7	1		0	0		2.5	4	
First Nations, Inuit, or Métis	0	0		0	0		0.5	1		0.6	1	
Hispanic	1.5	2		1.5	2		0.5	1		1.3	2	
Middle Eastern	0	0		0	0		0.5	1		4.5	7	
Other	1.5	2		0.7	1		0	0		1.9	3	
White	96.3	131		96.4	188		98.1	211		84.1	132	
Level of education												
High school or less	7.0	10		10.8	21		3.2	7		9.1	15	
Postsecondary (collegial/professional training)	44.4	63		43.1	84		30.5	66		43	71	
University	48.6	69		46.2	90		66.2	143		47.8	79	
Annual income ($Can)^a^												
Less than $35,000	35.8	46		9.3	18		6.1	13		5.7	9	
Between $35,000 and $54,999	39.6	51		10.8	21		6.1	13		7.6	12	
Between $55,000 and $74,999	19.4	25		14.9	29		15.4	33		8.9	14	
Between $75,000 and $94,999	3.9	5		27.3	53		24.3	52		34.2	54	
$95,000 or more	1.6	2		37.6	73		48.1	103		43.7	69	
Employment status												
Employed	54.7	76		69.2	135		53.0	114		95.2	157	
Student	0.0	0		1.5	3		3.7	8		1.8	3	
Preventive leave/at home	39.6	55		24.1	47		27.9	60		1.8	3	
Unemployed	5.7	8		5.2	10		15.3	33		1.2	2	
Marital status, in relationship	93.0	132		91.8	179		97.2	210		97.0	160	
Child sex, female	50.4	70		49.7	95		49.0	77		47.9	79	
Child age, mo	15.47	6.81	10-38	7.95	2.41	5-14				16.49	10.31	1-36
Childhood interpersonal trauma, CITI^b^	3.65	4.53	0-25	4.99	5.30	0-24	3.98	4.75	0-23	3.27	4.78	0-30
At least 4 traumas	38.3	54		49.0	95		38.0	82		29.7	49	
Childhood abuse or neglect (CTQ)^c^	31.92	9.87	25-81	36.37	14.32	25-96	32.77	11.29	25-95			
At least 1 type of abuse or neglect	26.9	38		35.1	66		22.7	49				
Physical abuse	7.0	10		9.4	18		6.5	14				
Sexual abuse	7.1	10		15.7	30		8.8	19				
Emotional abuse	16.2	23		24.0	46		13.0	28				
Physical neglect	13.4	19		19.3	37		10.2	22				
Emotional neglect	4.9	7		15.7	30		9.3	20				
General development	237.80	45.06	90-300	242.15	38.44	80-385	249.15	29.44	140-300			
Socioemotional developmental	0.61	0.45	0-2.44	3.26	1.62	0-8.17	0.70	0.33	0.22-2.07			
Negative affectivity	3.20	0.90	1.50-5.67	3.20	0.82	1.48-5.67	3.22	0.78	1.50-5.12	3.31	1.01	1.00-5.83

Note: CITI = Childhood Interpersonal Trauma Inventory.

a Personal income for study 1 and family income for studies 2 to 4.

b Participants are considered to have experienced significant traumas on the CITI when they endorsed 4 or more items.^18^

c Cut-offs for each subscale: physical abuse ≥8, psychological abuse ≥10, sexual abuse ≥8, physical neglect ≥8, and psychological neglect ≥15.^51^ Participants with at least 1 subscale with a score above the cutoff were classified as having been exposed to childhood abuse or neglect.

### Measures

#### Childhood Trauma

Two different measures of childhood trauma were used: the Childhood Trauma Questionnaire (CTQ),^52^ and the Childhood Interpersonal Trauma Inventory (CITI).^5^ Whereas the CTQ assesses experiences of abuse and neglect, the CITI captures a broader range of potentially traumatic experiences such as bullying, parentification, having a parent with a substance use disorder, having a parent who attempted suicide, parental alienation, or parental overprotection/overcontrol.^5^ The use of the latter instrument was prompted by findings from a recent study showing that the CITI can detect significant traumatic experiences that are not captured by the CTQ but that are nevertheless associated with poor outcomes in pregnant women.^5^ This dual assessment strategy in the current study allowed us to assess whether the intergenerational effects of childhood trauma are limited to abuse or neglect or whether they also apply to a broader range of interpersonal trauma.

More specifically, the CITI^5^ assesses adults’ cumulative exposure to childhood trauma. Participants are asked to identify whether or not they had been exposed before the age of 18 years to each of the 33 potentially traumatic experiences presented, using a yes/no format. Higher scores, derived by summing the number of situations that they experienced, indicate greater exposure to childhood trauma. The CITI has shown satisfactory convergent and incremental validity.^5^ In the current study, Cronbach alphas for the CITI were 0.88 in sample 1, 0.88 in sample 2, 0.88 in sample 3, and 0.90 in sample 4.

The Childhood Trauma Questionnaire Short Form (CTQ)^52^ was complementarily used in the 3 maternal samples at baseline but was not available in the sample of fathers. This 28-item self-report instrument assesses 5 types of childhood maltreatment: physical abuse, psychological abuse, sexual abuse, physical neglect, and emotional neglect. Each item is rated on a 5-point Likert scale. Total scores, derived by summing the responses, indicate greater exposure to childhood abuse and neglect. The CTQ has demonstrated strong validity across a variety of clinical and general populations.^52^ In the current study, Cronbach alphas for the CTQ were 0.80 in sample 1, 0.85 in sample 2, and 0.84 in sample 3.

#### General Development

The Ages and Stages Questionnaire, Third Edition (ASQ-3)^53^ was used to assess children's general development in the 3 samples of mother–child dyads. The ASQ-3 was not available in the sample of fathers. The appropriate version of the ASQ-3 was used according to the age of the child. This parent-reported questionnaire consists of 30 items describing child behavior, rated on a 3-point scale. It includes 5 subscales that screen for developmental delays in different domains (communication, gross motor skills, fine motor skills, problem solving, personal–social skills). Higher scores indicate more positive developmental outcomes. In this study, we aggregated scores from all age-specific versions into a unified variable, an approach that has been previously used.^54^ The ASQ-3 has robust psychometric properties.^53^ In the current study, Cronbach alphas for the ASQ-3 were 0.86 in sample 1, 0.78 in sample 2, and 0.77 in sample 3.

#### Socioemotional Difficulties

Socioemotional difficulties in children were rated by mothers in samples 1 through 3 using the Ages and Stages Questionnaire: Social–Emotional, Second Edition (ASQ:SE-2).^53^ The questionnaire was not available in the sample of fathers. The ASQ:SE-2 screens for socioemotional developmental difficulties in several domains (self-regulation, compliance, social communication, adaptive functioning, autonomy, affect, interaction with others) using a 3-point Likert scale. Higher scores on the ASQ:SE-2 indicate higher levels of difficulties in socioemotional development. This questionnaire includes specific versions tailored to different age intervals, with the number of items varying accordingly. Because the ages of the children in samples 1 and 2 varied, the age-appropriate version of the ASQ:SE-2 was used, and an aggregate score of social–emotional difficulties was calculated by merging the mean scores of the instruments. The ASQ:SE-2 has demonstrated good psychometrics properties.^55^ Cronbach alphas were calculated for versions of the ASQ:SE-2 administered to at least 45 participants.^56^ Cronbach alphas were 0.51 in sample 1, between 0.61 and 0.81 in sample 2, and 0.61 in sample 3, which is comparable to previous studies using a maternal report of child development.^57^

#### Negative Affectivity

Negative affectivity was assessed using the Short Form (25 items) of the Infant Behavior Questionnaire–Revised (IBQ-R SF)^58^ in samples 2 and 3 and the Very Short Form (12 items) of the IBQ-R^59^ or of the Early Childhood Behavior Questionnaire–Revised (ECBQ-R VSF),^59^ depending on the age of the child, in samples 1 and 4. Higher negative affectivity scores on these measures are indicative of the child expressing more frequent and intense negative emotions such as fear, frustration, sadness, and discomfort. In samples 1 and 4, in which 2 age versions were used, aggregate negative affectivity scores were calculated by merging mean scores, an approach used in previous studies.^60^ These questionnaires have strong psychometric properties.^58^^,^^59^ Cronbach alphas were respectively 0.76 (IBQ-VSF) and 0.75 (ECBQ-VSF) in sample 1, 0.88 in sample 2, 0.81 in sample 3, and 0.91 (IBQ-VSF) and 0.81 (ECBQ-VSF) in sample 4.

#### Potential Control Variables

Several characteristics of the parents and their children were considered as potential confounders in the analyses. Parents provided information on their age, level of education, annual income in Canadian dollars (personal income in sample 1 and family income in samples 2-4), and their child’s sex (assigned at birth) and age (in months).

### Data Analyses

Similar analyses were conducted across the 4 samples. An initial examination of bivariate correlations was conducted using SPSS 29.0 to identify potentially confounding variables to control for in the primary analyses. In each of the 4 statistical models and for each outcome, we controlled only for the variables that were significantly associated with this outcome in that particular sample (Tables S1-S4, available online). For the primary objectives, a regression-based statistical moderation analysis was conducted using the PROCESS macro^61^ in SPSS. This analysis tested for direct associations between parental childhood trauma and child outcomes, as well as the potential moderating effect of child sex. Two models were estimated for each child outcome: 1 model using the CITI (model 1) and 1 model using the CTQ (model 2). These analyses were conducted with 5,000 bootstraps. To limit the risk of bias, a Bonferroni correction was applied to account for multiple testing (3 child outcomes per sample in samples 1-3). Accordingly, *p* values were fixed at .017, and 98.3% CIs were computed for each analysis in samples 1 to 3, whereas *p* values of .05 and 95% CIs were used in sample 4, as only 1 outcome was assessed in this sample. If the CI did not include 0, the test was considered statistically significant.^61^ Because missing data followed a missing completely at random (MCAR) pattern in all samples (Supplement 1, available online), analyses were performed using only complete cases.

## Results

Tables S1 to S4, available online, show that child sex was not associated with sociodemographic characteristics in any of the 4 samples, but only in sample 3, where mothers of boys reported a lower income than mothers of girls. Annual family income was therefore included as a covariate in this model. The sociodemographic characteristics included as covariates in each statistical model are detailed in the footnotes of Table 2, Table 3, Table 4, Table 5.

**Table 2:** Results of the Regression-Based Moderation Analyses in Sample 1

Child outcomes		Predictor variables	*b* (SE)	β (SE)	*t*	*p*	98.3% CI
General development	Model 1	Level of education	4.69 (2.98)	0.14 (0.09)	1.57	.12	–0.08 to 0.36
development		Childhood interpersonal trauma	1.13 (1.09)	0.12 (0.12)	1.03	.30	–0.16 to 0.40
		Child sex	–11.59 (7.51)	–0.27 (0.17)	–1.54	.13	–0.68 to 0.15
		Childhood interpersonal trauma × child sex	–4.50 (1.65)	–0.48 (0.17)	–2.73	**.01**	–0.90 to –0.05
		Girls	1.13 (1.09)	0.12 (0.12)	1.03	.30	–0.16 to 0.40
		Boys	–3.37 (1.30)	–0.36 (0.14)	–2.60	**.01**	–0.69 to –0.02
	Model 2	Level of education	4.61 (2.93)	0.14 (0.09)	1.58	.12	–0.08 to 0.36
		Childhood abuse or neglect	–0.16 (0.49)	–0.04 (0.12)	–0.33	.74	–0.32 to 0.24
		Child sex	–10.63 (7.59)	–0.24 (0.17)	–1.40	.16	–0.67 to 0.18
		Childhood abuse or neglect × child sex	–0.95 (0.76)	–0.22 (0.18)	–1.26	.21	–0.65 to 0.21
Socioemotional difficulties	Model 1	Annual personal income	–0.04 (0.02)	–0.18 (0.10)	–1.88	.06	–0.41 to 0.05
		Age	–0.02 (0.01)	–0.20 (0.09)	–2.09	.04	–0.42 to 0.03
		Child age	0.02 (0.01)	0.30 (0.09)	3.31	**.001**	0.08 to 0.51
		Childhood interpersonal trauma	–0.01 (0.01)	–0.14 (0.12)	–1.15	.25	–0.42 to 0.15
		Child sex	0.05 (0.08)	0.12 (0.18)	0.66	.51	–0.32 to 0.55
		Childhood interpersonal trauma × child sex	0.04 (0.02)	0.41 (0.19)	2.20	.03	–0.04 to 0.86
	Model 2	Annual personal income	–0.04 (0.02)	–0.19 (0.10)	–2.02	.05	–0.42 to 0.04
		Age	–0.02 (0.01)	–0.16 (0.09)	–1.79	.08	–0.39 to 0.06
		Child age	0.02 (0.01)	0.32 (0.09)	3.62	**.001**	0.11 to 0.54
		Childhood abuse or neglect	–0.01 (0.01)	–0.16 (0.11)	–1.46	.15	–0.42 to 0.11
		Child sex	0.06 (0.08)	0.13 (0.18)	0.73	.47	–0.30 to 0.56
		Childhood abuse or neglect × child sex	0.02 (0.01)	0.36 (0.20)	1.86	.07	–0.11 to 0.84
Negative affectivity	Model 1	Childhood interpersonal trauma	0.01 (0.02)	0.06 (0.12)	0.48	.63	–0.23 to 0.34
		Child sex	–0.14 (0.16)	–0.16 (0.17)	–0.91	.37	–0.58 to 0.26
		Childhood interpersonal trauma × child sex	–0.01 (0.03)	–0.05 (0.18)	–0.29	.77	–0.47 to 0.38
	Model 2	Childhood abuse or neglect	–0.01 (0.01)	–0.10 (0.11)	–0.94	.35	–0.37 to 0.16
		Child sex	–0.13 (0.16)	–0.15 (0.17)	–0.85	.40	–0.57 to 0.27
		Childhood abuse or neglect × child sex	–0.004 (0.02)	–0.04 (0.18)	–0.22	.83	–0.47 to 0.39

Note: Model 1 was conducted with the Childhood Interpersonal Trauma Inventory (CITI). Model 2 was conducted with the Childhood Trauma Questionnaire (CTQ). A Bonferroni correction was applied to account for multiple testing (3 outcomes), and the p value was fixed at .017 (.05/3). Significant associations are highlighted in boldface type. Analyses on General development controlled for maternal education; analyses on Socioemotional development controlled for maternal age, maternal income, and child’s age; and analyses on Negative affectivity included no covariate (Table S1, available online). For the unstandardized solution, the CITI and CTQ variables were mean centered before computing the interaction term with the moderator to reduce multicollinearity among the predictor, interaction term, and moderator.^62^ This strategy helps to prevent inflated standard errors (and reduced statistical power) and to obtain reliable parameter estimates.^62^ Confidence intervals are reported for the standardized solution.

**Table 3:** Results of the Regression-Based Moderation Analyses in Sample 2

Child outcomes		Predictor variables	*b* (SE)	β (SE)	*t*	*p*	98.3% CI
General development	Model 1	Annual family income	2.89 (1.23)	0.19 (0.08)	2.35	.02	–0.01 to 0.38
Development		Childhood interpersonal trauma	–1.30 (0.79)	–0.18 (0.11)	–1.63	.10	–0.45 to 0.09
		Child sex	5.08 (5.81)	0.13 (0.15)	0.88	.38	–0.23 to 0.50
		Childhood interpersonal trauma × child sex	1.56 (1.08)	0.22 (0.15)	1.45	.15	–0.15 to 0.59
	Model 2	Annual family income	2.78 (1.26)	0.18 (0.08)	2.20	.03	–0.02 to 0.38
		Childhood abuse or neglect	–0.56 (0.33)	–0.21 (0.12)	–1.71	.09	–0.50 to 0.08
		Child sex	5.05 (5.95)	0.13 (0.15)	0.85	.40	–0.24 to 0.50
		Childhood abuse or neglect × child sex	0.38 (0.42)	0.14 (0.16)	0.91	.36	–0.23 to 0.52
Socioemotional difficulties	Model 1	Level of education	–0.05 (0.11)	–0.04 (0.08)	–0.45	.65	–0.24 to 0.17
		Annual family income	–0.13 (0.05)	–0.20 (0.08)	–2.43	**.016**	–0.41 to –0.002
		Child age	–0.08 (0.05)	–0.12 (0.07)	–1.63	.10	–0.29 to 0.06
		Childhood interpersonal trauma	0.07 (0.03)	0.23 (0.11)	2.05	.04	–0.04 to 0.49
		Child sex	–0.16 (0.23)	–0.10 (0.14)	–0.68	.50	–0.44 to 0.25
		Childhood interpersonal trauma × child sex	–0.08 (0.04)	–0.26 (0.14)	–1.81	.07	–0.61 to 0.09
	Model 2	Level of education	–0.04 (0.11)	–0.03 (0.09)	–0.39	.69	–0.24 to 0.17
		Annual family income	–0.16 (0.06)	–0.24 (0.09)	–2.77	**.01**	–0.45 to –0.03
		Child age	–0.08 (0.05)	–0.12 (0.07)	–1.57	.12	–0.29 to 0.06
		Childhood abuse or neglect	0.01 (0.01)	0.07 (0.12)	0.56	.58	–0.22 to 0.35
		Child sex	–0.17 (0.24)	–0.10 (0.15)	–0.71	.48	–0.46 to 0.25
		Childhood abuse or neglect × child sex	–0.01 (0.02)	–0.05 (0.15)	–0.31	.76	–0.40 to 0.31
Negative affectivity	Model 1	Childhood interpersonal trauma	0.05 (0.02)	0.35 (0.11)	3.13	**.002**	0.08 to 0.62
Affectivity		Child sex	–0.22 (0.12)	–0.27 (0.15)	–1.83	.07	–0.63 to 0.09
		Childhood interpersonal trauma × child sex	–0.05 (0.02)	–0.30 (0.15)	–2.03	.04	–0.67 to 0.06
	Model 2	Childhood abuse or neglect	0.01 (0.01)	0.21 (0.13)	1.68	.10	–0.09 to 0.51
		Child sex	–0.22 (0.13)	–0.27 (0.16)	–1.77	.08	–0.65 to 0.10
		Childhood abuse or neglect × child sex	–0.01 (0.01)	–0.12 (0.16)	–0.74	.46	–0.50 to 0.27

Note: Model 1 was conducted with the Childhood Interpersonal Trauma Inventory (CITI). Model 2 was conducted with the Childhood Trauma Questionnaire (CTQ). A Bonferroni correction was applied to account for multiple testing (3 outcomes), and the p value was fixed at .017 (.05/3). Significant associations are highlighted in boldface type. Analyses on General development controlled for family income; analyses on Socioemotional development controlled for family income, maternal education, and child’s age; and analyses on Negative affectivity included no covariate (Table S2, available online). For the unstandardized solution, the CITI and CTQ variables were mean centered before computing the interaction term with the moderator to reduce multicollinearity between the predictor, interaction term, and moderator.^62^ This strategy helps to prevent inflated standard errors (and reduced statistical power) and to obtain reliable parameter estimates.^62^ Confidence intervals are reported for the standardized solution.

**Table 4:** Results of the Regression-Based Moderation Analyses in Sample 3

Child outcomes		Predictor variables	*b* (SE)	β (SE)	*t*	*p*	98.3% CI
General development	Model 1	Annual family income	–0.63 (1.16)	–0.05 (0.09)	–0.54	.59	–0.26 to 0.16
development		Childhood interpersonal trauma	–1.02 (0.84)	–0.15 (0.13)	–1.21	.23	–0.46 to 0.15
		Child sex	–8.39 (4.94)	–0.29 (0.17)	–1.70	.09	–0.70 to 0.12
		Childhood interpersonal trauma × child sex	1.73 (1.12)	0.26 (0.17)	1.54	.13	–0.15 to 0.67
	Model 2	Annual family income	–0.27 (1.19)	–0.02 (0.09)	–0.22	.82	–0.23 to 0.19
		Childhood abuse or neglect	–0.50 (0.35)	–0.17 (0.12)	–1.45	.15	–0.46 to 0.11
		Child sex	–8.11 (4.90)	–0.28 (0.17)	–1.65	.10	–0.69 to 0.13
		Childhood abuse or neglect × child sex	1.06 (0.50)	0.36 (0.17)	2.14	.03	–0.05 to 0.77
Socioemotional difficulties	Model 1	Annual family income	–0.01 (0.01)	–0.04 (0.08)	–0.55	.59	–0.22 to 0.14
		Childhood interpersonal trauma	0.01 (0.01)	0.10 (0.12)	0.80	.42	–0.20 to 0.39
		Child sex	0.05 (0.04)	0.17 (0.15)	1.15	.25	–0.19 to 0.53
		Childhood interpersonal trauma × child sex	0.03 (0.01)	0.45 (0.15)	2.96	**.004**	0.08 to 0.82
		Girls	0.01 (0.01)	0.10 (0.12)	0.80	.42	–0.20 to 0.39
		Boys	0.04 (0.01)	0.55 (0.09)	5.84	**<.001**	0.32 to 0.78
	Model 2	Annual family income	–0.004 (0.01)	–0.03 (0.08)	–0.36	.72	–0.23 to 0.17
		Childhood abuse or neglect	0.01 (0.003)	0.18 (0.12)	1.54	.13	–0.10 to 0.47
		Child sex	0.05 (0.05)	0.18 (0.16)	1.15	.25	–0.20 to 0.56
		Childhood abuse or neglect × child sex	0.01 (0.005)	0.22 (0.16)	1.35	.18	–0.17 to 0.60
Negative affectivity	Model 1	Annual family income	0.06 (0.03)	0.16 (0.08)	2.02	.05	–0.03 to 0.36
		Childhood interpersonal trauma	0.03 (0.02)	0.20 (0.12)	1.59	.11	–0.10 to 0.49
		Child sex	–0.04 (0.12)	–0.05 (0.16)	–0.30	.76	–0.43 to 0.33
		Childhood interpersonal trauma × child sex	0.05 (0.03)	0.29 (0.16)	1.80	.07	–0.10 to 0.67
	Model 2	Annual family income	0.06 (0.03)	0.16 (0.09)	1.87	.06	–0.05 to 0.36
		Childhood abuse or neglect	0.01 (0.01)	0.15 (0.12)	1.21	.23	–0.15 to 0.45
		Child sex	–0.02 (0.12)	–0.03 (0.16)	–0.18	.86	–0.42 to 0.36
		Childhood abuse or neglect × child sex	0.02 (0.01)	0.23 (0.16)	1.37	.17	–0.17 to 0.62

Note: Model 1 was conducted with the Childhood Interpersonal Trauma Inventory (CITI). Model 2 was conducted with the Childhood Trauma Questionnaire (CTQ). A Bonferroni correction was applied to account for multiple testing (3 outcomes), and the p value was fixed at .017 (.05/3). Significant associations are highlighted in boldface type. All analyses controlled for annual family income, as it was not homogeneously distributed among mothers of boys and girls (Table S3, available online). For the unstandardized solution, the CITI and CTQ variables were mean centered before computing the interaction term with the moderator to reduce multicollinearity among the predictor, interaction term, and moderator.^62^ This strategy helps to prevent inflated standard errors (and reduced statistical power) and to obtain reliable parameter estimates.^62^ Confidence intervals are reported for the standardized solution.

**Table 5:** Results of the Regression-Based Moderation Analyses in Sample 4

Child outcomes	Predictor variables	*b* (SE)	β (SE)	*t*	*p*	95% CI
Negative affectivity	Childhood interpersonal trauma	0.06 (0.02)	0.28 (0.10)	2.72	**.01**	0.08 to 0.48
	Child sex	–0.32 (0.15)	–0.32 (0.15)	–2.08	**.04**	–0.62 to –0.02
	Childhood interpersonal trauma × child sex	–0.08 (0.03)	–0.36 (0.15)	–2.35	**.02**	–0.66 to –0.06
	Girls	–0.02 (0.02)	–0.08 (0.11)	–0.71	.48	–0.31 to 0.15
	Boys	0.06 (0.02)	0.28 (0.10)	2.72	**.01**	0.08 to 0.48

Note: Analyses were conducted with the Childhood Interpersonal Trauma Inventory (CITI). As only 1 outcome was measure in this sample, the p value was fixed at .05. No covariates were included in this model (Table S4, available online). Significant associations are highlighted in boldface type. For the unstandardized solution, the CITI variable was mean centered before computing the interaction term with the moderator to reduce multicollinearity among the predictor, interaction term, and moderator.^62^ This strategy helps to prevent inflated standard errors (and reduced statistical power) and to obtain reliable parameter estimates.^62^ Confidence intervals are reported for the standardized solution.

### Sample 1

The moderation analyses showed that child sex moderated the association between maternal childhood trauma, as measured by the CITI, and child general development (Table 2^62^). Specifically, the boys showed lower general development scores as the number of maternal childhood trauma experiences increased (Figure S1A, available online). No other results reached statistical significance.

### Sample 2

The moderation analyses showed that maternal childhood trauma, as measured by the CITI, was positively and significantly associated with levels of child negative affectivity (Table 3). No other results, including moderating effects, reached statistical significance.

### Sample 3

The moderation analyses showed that child sex moderated the association between maternal childhood trauma, as measured by the CITI, and socioemotional difficulties in the child (Table 4). Specifically, the boys exhibited higher socioemotional difficulties as their mothers reported more childhood trauma (Figure S1B, available online). No other associations were statistically significant.

### Sample 4

The moderation analyses showed that paternal childhood trauma was significantly and positively associated with negative affectivity in the child (Table 5). Results also showed that child sex moderated this association, with boys exhibiting higher negative affectivity as their fathers reported more childhood trauma (Table 5; Figure S1C, available online).

## Discussion

The aims of this study were to assess the associations between maternal and paternal childhood trauma and 3 indicators of early childhood functioning, and to examine whether the sex of the child moderated these associations. The study yields 4 major findings. First, in all 3 longitudinal studies of mother–child dyads, we observed prospective associations between maternal childhood trauma and at least 1 indicator of early childhood functioning among general development, socioemotional development, and negative affectivity. Second, we found novel findings linking paternal cumulative childhood trauma to negative affectivity in their child. Third, we found a moderating effect of child sex in 3 of the 4 samples, all indicating greater vulnerability for boys. Fourth, we observed that maternal cumulative exposure to interpersonal trauma during childhood was more predictive of offspring functioning than the severity of exposure to childhood abuse and neglect.

Our observation of a positive association between parents’ cumulative exposure to childhood trauma and at least one indicator of offspring functioning across the 4 samples is congruent with previous studies showing that interpersonal childhood trauma has intergenerational effects that can be observed very early in life.^7^^,^^13^^,^^28^^,^^30^ However, although an association between maternal childhood trauma and offspring functioning was found in the 3 samples that relied on maternal reports of child development, the specific domains affected differed across samples. Specifically, in the first sample, which included children assessed between 10 and 38 months postpartum, we observed an association between cumulative maternal childhood trauma and lower general development in boys. In the second sample, which included children between 5 and 14 months postpartum, we observed a significant association between maternal childhood trauma and increased negative affectivity. Finally, in the third sample, which included 6-month-old infants, maternal childhood trauma was associated with poorer socioemotional development in boys.

Numerous factors could have contributed to the observed discrepancies between samples involving mothers. Namely, the samples may have differed on important variables not controlled for in the analyses (eg, measures administered during or before the COVID-19 pandemic; paternal childhood trauma; time that the parent spends with the child), which could have played a significant role and modified the strength of the associations with maternal childhood trauma.^63^^,^^64^ In addition, the different age spans covered by the 3 samples of mother–child dyads may have contributed to these discrepancies. Indeed, children develop at an incredible pace during the first few years of life, and some vulnerabilities observed in infancy may normalize over time, whereas others may become more apparent as environmental demands on the child increase.^65^^,^^66^ Increased variance in scores due to rapid developmental changes at an earlier age can reduce the sensitivity of measures,^65^ thus making general developmental assessments less sensitive than specific measures, such as those focusing on socioemotional development and temperamental reactivity. This could explain why an association with general development was observed only in the older sample. In summary, the current study provides converging evidence for a potential intergenerational effect of maternal childhood trauma on domains of early offspring functioning; however, future longitudinal studies that follow children over time, assess different domains of child functioning at different ages, and measure other variables that may modulate the effect of maternal childhood trauma are needed to disentangle the complex associations between maternal childhood adversity and child functioning.

Importantly, with the exception of the association between maternal cumulative childhood trauma and children’s negative affectivity observed in the second sample, all associations between parental childhood trauma and early childhood functioning held only for boys. This finding is consistent with that of Letourneau *et al.*,^13^ who showed that, compared to young girls, young boys were more vulnerable to maternal childhood trauma in terms of internalizing and externalizing behaviors. The fact that our results showed a moderating effect of child sex very early in the child’s life suggests that biological mechanisms operating during pregnancy may be involved. Accordingly, male fetuses would have a lower placental reserve capacity and minimal placental adaptation in response to an adverse intrauterine environment compared with female fetuses.^45^^,^^46^ Consequently, their structural and functional intrauterine development may be particularly vulnerable to the effects of maternal stress during pregnancy and inflammation,^45^^,^^46^ which may result in an increased risk of adverse developmental outcomes.^67^ Accordingly, a previous study documented male fetal susceptibility to maternal inflammation, with higher levels of intrauterine C-reactive protein prospectively associated with lower levels of cerebral inhibition in newborn males,^67^ which in turn is involved in various cognitive, emotional, and behavioral functions^68^^,^^69^ and contributes to the risk of developing several mental health disorders.^70^ Intrauterine C-reactive protein has also been shown to predict poorer regulation/orientation in male but not female infants.^67^ Given that higher levels of inflammatory markers have been reported in trauma-exposed pregnant women than in nonexposed women,^71^ this male fetal susceptibility to maternal prenatal inflammation could potentially explain some of our findings.

Biological mechanisms may also be involved in the intergenerational effects of paternal childhood trauma. On the one hand, paternal childhood trauma has been associated with a higher risk of marital distress in childbearing partners,^72^ which may contribute to maternal stress and affect the intrauterine environment.^67^ On the other hand, epigenetic mechanisms cannot be excluded. Indeed, previous studies have reported that childhood trauma experienced by fathers is biologically transmitted to their children through epigenetic changes related to stress response and development, independent of socioeconomic variables, maternal childhood trauma, and maternal cortisol levels during pregnancy.^73^

Another explanation for the greater vulnerability of boys to the intergenerational effects of parental childhood trauma may be related to the quality of parent–child interactions. Indeed, numerous studies have shown an association between exposure to childhood trauma and hostile, intrusive, harsh, withdrawn, or atypical parenting behaviors,^74^^,^^75^ which in turn increase the risk of internalizing and externalizing problems in children.^76^ Previous studies have intriguingly suggested that boys may be more sensitive than girls to environmental factors such as parental behaviors,^47^^,^^48^ which may render them more vulnerable to developmental and socioemotional difficulties in response to negative parenting behaviors. Overall, although the current study provides strong support for male vulnerability to parental childhood trauma during early development, the exact mechanisms involved remain uncertain, and future studies are needed to elucidate the contribution of biological, epigenetic, interpersonal, and social factors in the intergenerational effects of parental childhood trauma on boys’ development and functioning during infancy and toddlerhood.

Another important finding of the current study is the unexpected observation that only parental retrospective reports of cumulative exposure to childhood interpersonal trauma, as measured by the CITI, yielded significant associations with child outcomes, whereas the severity of exposure to abuse and neglect, as measured by the CTQ, did not. Unlike the CTQ, which focuses on child maltreatment, the CITI includes a broader range of potentially traumatic childhood experiences.^5^ Two reasons may help explain why child-related outcomes were associated only with CITI scores and not with CTQ scores. First, the CITI captures cumulative exposure to multiple types of childhood trauma,^5^ whereas the CTQ assesses the frequency of exposure to 5 types of abuse or neglect.^51^ In this regard, previous studies have shown that the co-occurrence of different types of childhood trauma may have more drastic effects on development than the severity of exposure to any 1 type of childhood trauma.^77^ Second, not assessing a wide range of potentially traumatic experiences increases the risk of “contamination” in trauma research, which occurs when participants who score low on measures of childhood trauma have been exposed to experiences similar to those of participants who score high on the measure.^78^ Overall, this finding calls for broadening the focus beyond abuse and neglect when studying the intergenerational effects of childhood trauma.

The current study has several strengths. First, similar analyses were performed in 3 different samples of mothers and 1 sample of fathers. Second, this is among the first studies to assess the intergenerational effects of paternal childhood trauma. Third, a variety of child outcomes were included, which allows for the consideration of multiple facets of child functioning and thus provides a more comprehensive picture of the intergenerational effects of childhood trauma. Fourth, 2 complementary validated measures of childhood trauma were used, which increased the power to detect a potential effect of childhood adversity. However, certain methodological limitations should be considered. First, our samples are not ethnically and socioeconomically diverse, which limits generalizability. Second, the use of retrospective self-report instruments to assess childhood trauma and parent reports to assess child functioning increases the risk of response bias and shared method variance. In addition, the internal consistency of some instruments assessing child functioning was low, which may have affected the capacity to detect associations. Our findings thus call for future studies using observational measures of child development and temperament. Third, sample sizes were relatively small. Fourth, the use of different versions of the IBQ to measure infant negative affectivity may have contributed to some degree of inconsistency across samples.

The results from the present study have implications for clinical practice. First, the findings call for a broader screening of childhood traumatic life events among parents, as only cumulative exposure to different types of potentially traumatic experiences was predictive of offspring functioning, not the severity of exposure to abuse and neglect. Second, the findings call for the implementation of interventions aimed at mitigating the intergenerational effects of childhood trauma. In this vein, 2 prenatal interventions have been developed for women who have experienced childhood trauma.^79^, ^80^, ^81^ Future studies should evaluate whether these prenatal interventions contribute to mitigating the effect of maternal childhood trauma on boys’ early development and functioning.

In conclusion, infants and toddlers of parents who have experienced interpersonal trauma during their childhood are at risk for poorer general development, poorer socioemotional development, and a temperament characterized by negative affectivity, with boys being particularly vulnerable. Further studies are needed to better understand the mechanisms underlying the sex-specific intergenerational effects of childhood trauma. This could lead to the emergence of a new model of personalized medicine in perinatal practice that could allow intervention with the right person at the right time.

## CRediT authorship contribution statement

**Karl Larouche:** Writing – review & editing, Writing – original draft, Validation, Software, Project administration, Methodology, Investigation, Funding acquisition, Formal analysis, Data curation, Conceptualization. **Julia Garon-Bissonnette:** Writing – review & editing, Project administration, Methodology, Data curation, Conceptualization. **Roxanne Lemieux:** Writing – review & editing, Methodology, Conceptualization. **Kim Deschênes:** Writing – review & editing, Project administration, Methodology. **Gabrielle Duguay:** Writing – review & editing, Project administration, Methodology, Conceptualization. **Jean-Pascal Lemelin:** Writing – review & editing, Methodology. **Nicolas Berthelot:** Writing – review & editing, Validation, Supervision, Resources, Methodology, Investigation, Funding acquisition, Conceptualization.
