# Supplementary material for: Converging Evidence of a Specific Vulnerability of Young Boys to Parental Childhood Trauma
Source: JAACAP Open. 2025 Apr 2;3(4):1147–60. doi: 10.1016/j.jaacop.2025.03.001 (PMC12684472; doi:10.1016/j.jaacop.2025.03.001)
Supplement: Supplement Material [file mmc1.docx]

**Electronic supplement to the article Converging evidence of a specific vulnerability of young boys to parental childhood trauma**

**Supplement 1**

Data were missing for the following variables in sample 1: mothers’ age (0.7%), level of education (0.7%), personal annual income (9.8%), depressive symptoms (0.7%), childhood interpersonal trauma (CITI; 1.4%), childhood abuse and neglect (CTQ; 1.4%), general development (6.3%), socio-emotional development (3.5%), negative affectivity (2.1%), child sex (2.8%), and child age (7.7%). The missing data followed an MCAR pattern (*χ^2^* [116] = 139.92, *p* = 0.07). In sample 2, data were missing for the following variables: mothers’ age (0.5%), family annual income (0.5%), psychological distress (3.1%), childhood interpersonal trauma (CITI; 0.5%), childhood abuse and neglect (CTQ; 3.6%), general development (11.8%), socioemotional development (3.1%), negative affectivity (9.2%), child sex (2.1%), and child age (2.6%). The missing data followed an MCAR pattern (*χ^2^* [162] = 186.56, *p* = 0.09). In sample 3, data were missing for the following variables: mothers’ family annual income (0.9%) and prenatal psychological distress (0.5%), general development (7.4%), socioemotional development (2.3%), negative affectivity (4.6%), and child sex (27.3%). The missing data followed an MCAR pattern (*χ^2^* [66] = 84.36, *p* = 0.06). Finally, in sample 4, data were missing for the following variables: fathers’ age (0.6%), family annual income (4.2%), and negative affectivity (1.2%). The missing data followed a MCAR pattern (*χ^2^* [27] = 36.90, *p* = 0.10). As missing data followed an MCAR pattern for samples 1 to 4, all analyses were conducted using complete cases.

**Table S1. Correlations of Potential Control Variables and Main Variables in Sample 1**

|  | 2 | 3 | 4 | 5 | 6 | 7 | 8 | 9 | 10 |
| --- | --- | --- | --- | --- | --- | --- | --- | --- | --- |
| 1. Age | .05 | .29** | -.06 | -.06 | -.15 | -.11 | .00 | -.18* | -.03 |
| 2. Level of education |  | .62** | -.04 | .06 | -.29** | -.18** | .19* | -.12 | .10 |
| 3. Annual personal income |  |  | -.02 | -.15* | -.27** | -.28** | .18 | -.27** | -.01 |
| 4. Child sex (boy) |  |  |  | .12 | .06 | -.04 | -.12 | .15 | -.001 |
| 5. Child age |  |  |  |  | -.03 | .02 | .03 | .32** | -.08 |
| 6. Childhood interpersonal trauma (CITI) |  |  |  |  |  | .69** | -.08 | .09 | .05 |
| 7. Childhood abuse or neglect (CTQ) |  |  |  |  |  |  | -.13 | .002 | -.11 |
| 8. General development |  |  |  |  |  |  |  | -.16 | .27** |
| 9. Socioemotional difficulties |  |  |  |  |  |  |  |  | .29** |
| 10. Negative affectivity |  |  |  |  |  |  |  |  |  |

**Table S2. Correlations of Potential Control Variables and Main Variables in Sample 2**

|  | 2 | 3 | 4 | 5 | 6 | 7 | 8 | 9 | 10 |
| --- | --- | --- | --- | --- | --- | --- | --- | --- | --- |
| 1. Age | .18* | .14 | .03 | .03 | -.15* | -.10 | .06 | -.02 | .05 |
| 2. Level of education |  | .51** | -.05 | .09 | -.34** | -.32** | .09 | -.17* | -.10 |
| 3. Annual family income |  |  | -.03 | .10 | -.33** | -.31** | .22** | -.25** | -.12 |
| 4. Child sex (boy) |  |  |  | -.08 | .09 | .13 | .05 | -.02 | -.12 |
| 5. Child age |  |  |  |  | -.08 | -.04 | .01 | -.15* | .03 |
| 6. Childhood interpersonal trauma (CITI) |  |  |  |  |  | .79** | -.13 | .17* | .17* |
| 7. Childhood abuse or neglect (CTQ) |  |  |  |  |  |  | -.18* | .13 | .11 |
| 8. General development |  |  |  |  |  |  |  | -.26** | -.01 |
| 9. Socioemotional difficulties |  |  |  |  |  |  |  |  | .34** |
| 10. Negative affectivity |  |  |  |  |  |  |  |  |  |

**Table S3. Correlations of Potential Control Variables and Main Variables in Sample 3**

|  | 2 | 3 | 4 | 5 | 6 | 7 | 8 | 9 |
| --- | --- | --- | --- | --- | --- | --- | --- | --- |
| 1. Age | .29** | .24** | -.09 | .01 | .09 | -.04 | -.08 | .11 |
| 2. Level of education |  | .35** | -.06 | -.25** | -.22** | -.04 | -.08 | .11 |
| 3. Annual family income |  |  | -.22** | -.17* | -.20** | -.07 | -.08 | -.01 |
| 4. Child sex (boy) |  |  |  | .07 | .05 | -.14 | .12 | -.03 |
| 5. Childhood interpersonal trauma (CITI) |  |  |  |  | .84** | -.02 | .40** | .27** |
| 6. Childhood abuse or neglect (CTQ) |  |  |  |  |  | .03 | .35** | .18* |
| 7. General development |  |  |  |  |  |  | -.10 | .03 |
| 8. Socioemotional difficulties |  |  |  |  |  |  |  | .44** |
| 9. Negative affectivity |  |  |  |  |  |  |  |  |

**Table S4. Correlations of Potential Control Variables and Main Variables in Sample 4**

|  | 2 | 3 | 4 | 5 | 6 | 7 |
| --- | --- | --- | --- | --- | --- | --- |
| 1. Age | .01 | -.01 | .07 | .16* | .06 | -.10 |
| 2. Level of education |  | .30** | .004 | -.03 | -.17* | -.03 |
| 3. Annual family income |  |  | .07 | -.10 | -.04 | -.12 |
| 4. Child sex (boy) |  |  |  | .002 | .04 | .16* |
| 5. Child age |  |  |  |  | .02 | -.01 |
| 6. Childhood interpersonal trauma (CITI) |  |  |  |  |  | .12 |
| 7. Negative affectivity |  |  |  |  |  |  |

**Figure S1. Moderating Effect of Child Sex on the Associations Between Parental Trauma and Child Functioning**

Figure S1C

Figure S1B

Figure S1A

***Note****:* Figure S1A = Sample 1 (N = 143), Figure S1B = Sample 3 (N = 216), and Figure S1C = Sample 4 (N = 165).
